# Dr. James C. Culbertson

**Published:** 1908-07

**Authors:** 


### OBITUARY

Dr. James C. Culbertson, of Cincinnati, died at his home in that city June 4, 1908, after a long illness, from arteriosclerosis, aged sixty-seven years. He was editor and publisher of the *Cincinnati Lancet-Clinic* for thirty years and for two years, from 1891 to 1893, editor of the *Journal of the American Medical Association.* He served in the Civil War, first as a private soldier, then as hospital steward and finally as assistant surgeon of the 137th Ohio volunteer infantry. He was professor of medicine in the Cincinnati College of Medicine and Surgery and prominent in civil affairs of his city. He was a companion of the Military Order of the Loyal Legion of the United States, being a member of the Ohio commandery.

